# Research- vs. government-driven physical activity policy monitoring: a systematic review across different levels of government

**DOI:** 10.1186/s12961-023-01068-5

**Published:** 2023-11-27

**Authors:** Sven Messing, Antonina Tcymbal, Karim Abu-Omar, Peter Gelius

**Affiliations:** 1 Department of Sport Science and Sport, Friedrich-Alexander-Universität Erlangen-Nürnberg, Erlangen, Germany; 2 Institute of Sport Sciences, Université de Lausanne, Lausanne, Switzerland

**Keywords:** Public health, Physical activity, Policy, Monitoring, Methodology, Research-policy relations

## Abstract

**Background:**

Even though the importance of physical activity policy monitoring has increased in the last decade, there is a lack of understanding what different approaches exist and which methodology they employ. In order to address this research gap, this review attempts to map existing approaches of physical activity policy monitoring and to analyse methodological aspects, especially with regards to the roles of governments and researchers.

**Methods:**

A systematic search was conducted in five scientific databases (PubMed, Scopus, SportDiscus, Psycinfo, Web of Knowledge) in July 2021, and the identified records were screened independently by two reviewers. Records were included if they (a) focused on the monitoring of public policies to promote PA, (b) allowed to compare policies across time, across nations/regions or across policy sectors, and (c) were written in English, German or Russian. During full text analysis, information on methodological aspects was extracted and studies were categorized based on the level of government involvement.

**Results:**

The search yielded in a total of 112 studies. 86 of these studies (76.8%) followed a research-driven approach (little or no government involvement) while only two studies (1.8%) were based on a government-driven approach (led by governments). The remaining 24 studies (21.4%) were based on a co-production approach (strong collaboration between researchers and governments). All in all, 18 different tools for physical activity policy monitoring were identified; key examples are the Report Cards on Physical Activity for Children and Youth (research-driven approach), the HEPA Monitoring Framework (government-driven approach) and the HEPA Policy Audit Tool (co-production approach).

**Conclusions:**

The level of government involvement in policy monitoring differs significantly, and research-driven, government-driven and co-production approaches can be distinguished. These approaches have different strengths and weaknesses, and can be linked to distinct theories of change and models on research-policy relations. Increasing awareness on the implications of these approaches is key to improve the understanding and further development of physical activity policy monitoring.

**Supplementary Information:**

The online version contains supplementary material available at 10.1186/s12961-023-01068-5.

## Background

Physical inactivity is a global problem causing more than 5.3 million premature deaths per year worldwide [1], and studies have shown that 27.5% of adults and 81.0% of adolescents are inactive [2, 3]. If inactivity were decreased by 25%, more than 1.3 million deaths could be averted each year [1]. Consequently, the World Health Organisation (WHO) set a target of a 15% reduction in global physical inactivity by 2030 [4]. From a public health perspective, it is becoming increasingly clear that physical activity (PA) promoting policies are needed to combat inactivity, and WHO highlighted the necessity of public policies to achieve their target [4]. Also, the Council of the European Union has recommended the development of public policies to promote PA across different political sectors such as sport, health, education, transport and workplace [5]. In addition, there is a growing body of evidence on the effectiveness of PA promoting policies [6–10].

In addition to studies on the effectiveness of policies, a systematic and ideally regular collection of data on existing public policies on PA promotion seems to be highly relevant, as this would allow for comparing policies across time, nations or regions, and sectors. Such policies might include formal or informal legislative or regulatory action, statements of intent, or guides to actions issued by governments or organisations [7, 11–14]. The Organisation for Economic Cooperation and Development (OECD) defines policy monitoring as “continuous process of collecting and analyzing data to compare how well a project, program or policy is being implemented against expected results” [15, 16]. To date, a couple of highly visible approaches are established in the field of PA policy monitoring. Prominently, the European Commission developed a framework to monitor the implementation of the Council Recommendations on promoting Health Enhancing Physical Activity (HEPA) across sectors [17] in all 27 EU member states on a triennial basis. Other well-known tools have been developed by WHO (Health-Enhancing Physical Activity Policy Audit Tool, HEPA PAT, [18]) and the non-governmental organization Active Healthy Kids Global Alliance (Report Card on Physical Activity for Children and Youth, [19]). Additionally, researchers have recently developed a number of new approaches to monitor PA promoting policies (e.g., [20–22]).

However, due to the rapid development of this field, there is a gap of knowledge on what approaches for PA policy monitoring exist and which methodology they employ. This information is essential since policy monitoring, as health behaviour surveillance, is seen as informing and potentially impacting policymaking itself. In this context, theories of change in social systems [23] are of relevance, as they raise the question how research leads to change (e.g., whether policymakers should be involved from the very beginning to build capacity or whether it is sufficient to present results at the end of the research process). This links very well to models of research-policy relations [24], as they can help us to understand the impact of policy monitoring on the policy-making process. Boswell & Smith (2017) identified four models of research-policy relations that might help to differentiate between research- and government-driven approaches of PA policy monitoring:

1. Knowledge shapes policy: From this perspective, there is a gap between research and policy communities, and the impact of research is often reduced by problems of communication.
2. Politics shape knowledge: This perspective highlights the influence of politics on research, e.g. by commissioning research directly or indirectly from government sources or by a higher likeliness of research that supports dominant political interests to be employed in policymaking.
3. Co-production: The idea of co-production is based on the claim that knowledge and governance are mutually constitutive and influence each other.
4. Autonomous spheres: In contrast to the previous models, this perspective conceptualizes research and politics as distinct spheres that operate according to a separate logic.

In the field of PA policy monitoring, several of these models seem to exist but these differences have not been investigated yet. For this reason, the study aims (a) to map existing approaches of PA policy monitoring, and (b) to analyse methodological aspects of PA policy monitoring, with a specific focus on (c) discussing differences of research- vs. government driven approaches to monitor PA policies. To date, research in this field focuses mainly on the results of PA policy monitoring activities (e.g., [25]), describes the development of new tools (e.g., [18]) or identifies scientific instruments for analysing national-level PA policies [26]. This study complements existing research by its unique focus on methodological aspects and research-policy relations in the field of PA policy monitoring.

## Methods

### Information sources and search strategy

The literature search followed the Preferred Reporting Items for Systematic Review and Meta-Analysis (PRISMA) guidelines [27, 28]. In order to identify studies that monitor policies for PA promotion, a systematic search was conducted in five electronic databases (PubMed, Scopus, SportDiscus, Psycinfo, Web of Knowledge) in July 2021. Search terms were ‘physical activity’, ‘policy’ and ‘monitoring’ and respective alterations of each term (Additional file 1). No restrictions on language or publication date were applied.

### Eligibility criteria

**Fig. 1:**
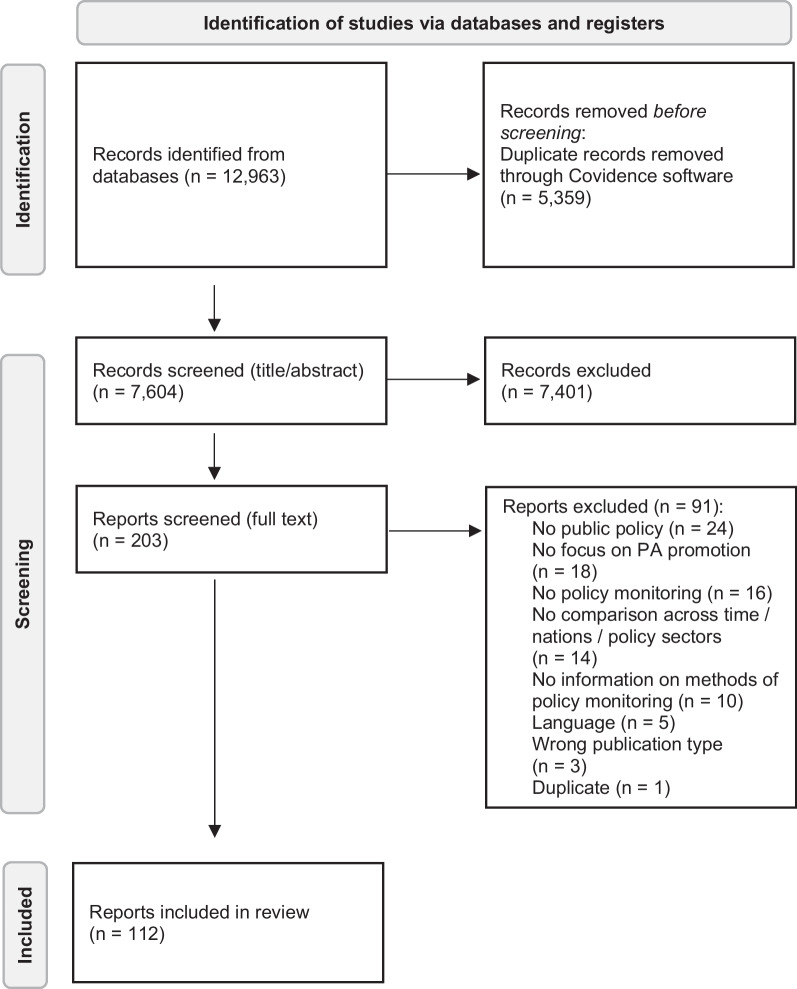
Flow chart

The screening process was based on three inclusion criteria: (a) The study focuses on the monitoring of public policies to promote PA, (b) the study design allows to compare policies across time, across nations/regions or across policy sectors, and (c) the study language is English, German or Russian. Records that did not focus primarily on PA promotion, intervention studies that were not related to public policies, and case studies that did not allow to compare policies across time, across nations/regions or across policy sectors were excluded (Fig. 1).

### Study selection

Duplicates were excluded automatically using the software Covidence (Veritas Health Innovation, Melbourne, Australia; www.covidence.org). Titles and abstracts of identified records were screened by five reviewers, with each study being assessed by two of these reviewers independently. Disagreements were resolved by a consensus-based discussion. The remaining full texts were screened by two reviewers independently, with disagreements being solved in consensus. Intercoder reliability was 97.0% for title and abstract screening and 83.3% for full text screening.

### Data extraction

For data extraction, studies were categorized in an inductive process based on their key characteristics. Data extraction was divided between four reviewers, with each category being double-checked by a second reviewer to ensure the consistency of the process. The process was guided by a data extraction template that focused mainly on methodological aspects of the policy monitoring process described in each study (policy monitoring tool, policy level, no. of countries, frequency, systematic search, expert consultation, consultation method, document analysis, level of detail, purpose, assessment method, government involvement, monitoring approach). We distinguished between the purpose of auditing and assessing PA policies: While auditing is a “prerequisite for policy assessment”, the assessment includes additionally a grading, rating, judging or evaluation of policies [29].

### Data synthesis

Based on theoretical considerations grounded in theories of change [23] and research-policy relations [24], the level of government involvement was considered to be a key feature of policy monitoring. After extracting data on whether and to what extent governmental actors were involved in the monitoring process, the monitoring approach was assessed on a five-tier scale (purely research-driven, mainly research-driven, co-production, mainly government-driven, purely government-driven). This allowed to differentiate between monitoring approaches purely or mainly led by scientists (“knowledge shapes policy”; e.g., systematic reviews, desk research, surveys), processes that require a close collaboration of scientists and governments (“co-production”), and monitoring approaches purely or mainly led by governments (“policy shapes knowledge”; e.g., supranational policy monitoring initiated by EU institutions).

## Results

### Study selection process

During the search in five databases, 12.963 records were identified. After the removal of duplicates, the remaining 7.604 records were screened based on titles and abstracts. The search yielded in a total of 112 reports of unique studies (Fig. 1).

### Overview of included studies

The key features of the included studies are presented in Table 1 (a list of all included studies is available as Additional file 2). 85 of the included 112 studies monitored national level policies (75.9%), and 78 focused on a single country (69.6%). 58 studies were part of approaches to regular policy monitoring (51.8%), and 60 studies had the purpose to assess policies (53.6%). 86 studies consulted experts (73.8%), 69 conducted systematic searches (61.6%) and 89 analysed the content of policy documents (79.5%).

**Table 1:** Key features of approaches for policy monitoring

Key features	Answer options	Report Cards	HEPA Monitoring Framework	HEPA PAT	Other	Total
Policy level^a^	Inter-/supranational	0.0% (0/47)	0.0% (0/5)	0.0% (0/3)	3.5% (2/57)	1.8% (2/112)
	National	87.2% (41/47)	100.0% (5/5)	100.0% (3/3)	63.2% (36/57)	75.9% (85/112)
	Regional	12.8% (6/47)	0.0% (0/5)	0.0% (0/3)	50.9% (29/57)	31.3% (35/112)
	Local	0.0% (0/47)	0.0% (0/5)	0.0% (0/3)	8.8% (5/57)	4.5% (5/112)
No. of countries	10 or more	0.0% (0/47)	100.0% (5/5)	0.0% (0/3)	21.1% (12/57)	15.2% (17/112)
	2–9	0.0% (0/47)	0.0% (0/5)	100.0% (3/3)	24.6% (14/57)	15.2% (17/112)
	1	100.0% (47/47)	0.0% (0/5)	0.0% (0/3)	54.4% (31/57)	69.6% (78/112)
Frequency	Regular monitoring	100.0% (47/47)	100.0% (5/5)	0.0% (0/3)	10.5% (6/57)	51.8% (58/112)
	Single study	0.0% (0/47)	0.0% (0/5)	100.0% (3/3)	89.5% (51/57)	48.2% (54/112)
Purpose of analysis	Auditing	0.0% (0/47)	100.0% (5/5)	100.0% (3/3)	77.2% (44/57)	46.4% (52/112)
	Assessment	100.0% (47/47)	0.0% (0/5)	0.0% (0/3)	22.8% (13/57)	53.6% (60/112)
Monitoring approach	Purely research-driven	55.3% (26/47)	0.0% (0/5)	0.0% (0/3)	52.6% (30/57)	50.0% (56/112)
	Mainly research-driven	8.5% (4/47)	20.0% (1/5)	66.7% (2/3)	40.4% (23/57)	26.8% (30/112)
	Co-production	36.2% (17/47)	40.0% (2/5)	33.3% (1/3)	7.0% (4/57)	21.4% (24/112)
	Mainly government-driven	0.0% (0/47)	40.0% (2/5)	0.0% (0/3)	0.0% (0/57)	1.8% (2/112)
	Purely government-driven	0.0% (0/47)	0.0% (0/5)	0.0% (0/3)	0.0% (0/57)	0.0% (0/112)
Government involvement	Yes	44.7% (21/47)	100.0% (5/5)	100.0% (3/3)	47.4% (27/57)	50.0% (56/112)
	No	55.3% (26/47)	0.0% (0/5)	0.0% (0/3)	52.6% (30/57)	50.0% (56/112)
Expert consultation	Yes	100.0% (47/47)	100.0% (5/5)	100.0% (3/3)	54.4% (31/57)	76.8% (86/112)
	No	0.0% (0/47)	0.0% (0/5)	0.0% (0/3)	45.6% (26/57)	23.2% (26/112)
Systematic search	Yes	59.6% (28/47)	40.0% (2/5)	100.0% (3/3)	63.2% (36/57)	61.6% (69/112)
	No	40.4% (19/47)	60.0% (3/5)	0.0% (0/3)	36.8% (21/57)	38.4% (43/112)
Document analysis	Yes	78.7% (37/47)	80.0% (4/5)	100.0% (3/3)	78.9% (45/57)	79.5% (89/112)
	No	21.3% (10/47)	20.0% (1/5)	0.0% (0/3)	21.1% (12/57)	20.5% (23/112)
Total		100.0% (47)	100.0% (5)	100.0% (3)	100.0% (57)	100.0% (112)

^a^Multiple answer categories possible

**Fig. 2:**
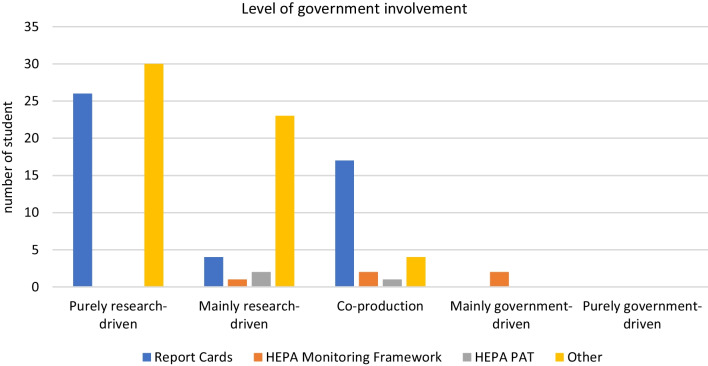
Level of government involvement

The government was involved in 56 of the included studies (50.0%). Based on the level of government involvement, the studies were sorted into the five different categories (Fig. 2):

- 56 studies (50.0%) were categorised as following a purely research-driven approach with no involvement of government officials. In this category, data collection was mainly based on (systematic) reviews of the scientific literature (e.g., [30, 31]), database or website searches (e.g., [32, 33]), or a combination of both.
- 30 studies (26.8%) applied a mainly research-driven approach. Most of these studies involved government officials in data collection, especially via expert interviews (e.g., [34, 35]) or surveys (e.g., [36, 37]). In some studies, government officials were also involved in data validation (e.g., [38, 39]).
- 24 studies (21.4%) utilized co-production approaches that initiated close collaboration between researchers and government officials, especially for data collection (e.g., [40]). In some of these studies, an additional feedback loop took place at the end of the process to consolidate and validate the findings [21, 41].
- 2 studies (1.8%) utilized a mainly government-driven approach. Both studies are based on the EU/WHO HEPA Monitoring Framework and are described in detail in the respective section of this manuscript.
- No study (0.0%) used a purely government-driven approach.

### Tools for physical activity policy monitoring

73 of the 112 studies (65.2%) utilized a specific tool for policy monitoring, and a total of 18 different tools for PA policy monitoring were identified (Table 2). The most frequently applied tools were the Report Card on Physical Activity for Children and Youth, the EU/WHO HEPA Monitoring Framework and the Health-Enhancing Physical Activity Policy Audit Tool (HEPA PAT). These different tools and their perspectives on PA policy monitoring are described in the following sections of this manuscript.

**Table 2:** Identified tools for policy monitoring

Tool	Short description	No. of included studies*
*General tools*		
Report Card on Physical Activity for Children and Youth	Grading of indicators relevant for the physical activity behaviour of children and adolescents, including an indicator on “government”	56
EU/WHO HEPA Monitoring Framework	Monitoring of the implementation of the EU Council Recommendations on HEPA, consisting of 23 indicators in 10 thematic areas such as sport, health, and education	5
Health-Enhancing Physical Activity Policy Audit Tool (HEPA PAT)	Auditing of national policy approaches to PA promotion, consisting of 29 closed and open-ended questions in 10 sections	3
Active Community Environments (ACE)	15-question survey to assess counties’ policies related to six domains (sidewalks, bike lanes, greenways, recreational facilities, commercial buildings, share-use paths) [42, 43]	2
Global Observatory for Physical Activity (GoPA!)	Measuring global progress in the areas of surveillance, policy, and research [39]; The Global Observatory for Physical Activity-GoPA! Policy Inventory, version 3.0 [44]	2
Analysis Grid for Environments Linked to Obesity (ANGELO) Framework	Framework for mapping broad portfolios of interventions [45], developed by Swinburn et al. [46]	1
Australian Systems Approach to Physical Activity (ASAPA) tool	Identification of policy content according to a defined set of criteria [21]	1
CAPLA-Santé	Auditing of local policy approaches to PA, development based on the HEPA PAT version 2 [20], developed by Racine et al. [47]	1
Community Health Assessment and Group Evaluation (CHANGE)	Auditing and assessing policy and environmental changes in communities [40]	1
Eurostat	Data on government expenditure on sports and recreation [33]	1
Four cornerstones of a successful national policy framework	Identification of four key areas of policy [48]	1
HARDWIRED	Nine criteria for successful national physical activity policy [49]	1
WHO NCD progress monitor report	Assessment of policies to prevent noncommunicable diseases (NCDs) and their alignment to the best buys [50]	1
*School setting*		
School Health Policies and Programs Study (SHPPS)	Assessing school health programs and policies [51]	1
School Physical Activity Policy Assessment (S-PAPA)	Assessing direct and school level PA policy, related school environmental variables, and policy implementation at a school site [52]	1
Wellness School Assessment Tool	Evaluating school districts’ efforts regarding physical education and physical activity in school [53]	1
Worldwide Survey of the situation of physical education in schools	Assessing the worldwide situation of physical education in schools [54]	1
*Childcare setting*		
Health Eating and Physical Activity (HEPS) inventory tool	Quality assessment of school interventions on healthy eating and physical activity [55]	1

### Report card on physical activity for children and youth: “knowledge shapes policy”

47 studies utilized the Report Card on Physical Activity for Children and Youth, a tool that grades PA behaviour of children and adolescents as well as related organisational and public policies within a country [19, 56]. National Report Cards are issued in intervals of two to four years, and country-specific results are compared in a Global Matrix; the current Global Matrix 4.0 is implemented in 57 countries on six continents [56]. However, only one out of 10 indicators that Report Cards contain refers to public policies of national governments.

Most of the studies in this category focus on the national level (87.2%); only in the United Kingdom, the tool was applied at regional level to develop specific Report Cards for the three home countries England, Scotland, and Wales [57–59]. Less than half of all Report Card studies involved government officials (44.7%). However, the level of government involvement differed: While government officials were part of the Research Work Group in some countries [60, 61], other countries involved representatives of their government in single stages of the process such as data collection [62] or grade assignment [63]. The approaches applied in the Report Card studies were classified as being purely or mainly research-driven or—if government representatives were part of the respective Research Work Groups—as co-productive.

### HEPA Monitoring Framework: “policy shapes knowledge”

Five studies were based on the HEPA Monitoring Framework that aims to monitor the implementation of the EU Council Recommendation on Health Enhancing Physical Activity [5, 64]. The monitoring framework is based on a staff working document of the European Commission, and consists of 23 indicators in ten thematic areas such as sport, health, and education [17]. The monitoring itself is supported by the WHO Regional Office for Europe [64].

All studies in this category focus on national level policies and usually include data from all member states of the European Union. The level of government involvement is high, as data are collected through a triennial survey among the national HEPA focal points who are appointed by each EU Member State [64]. During data collection, WHO maintained a helpdesk, conducted several webinars with focal points, reviewed and validated responses, and followed up with focal points where further information was required [25]. The HEPA Monitoring Framework is a precedent for a government-driven approach as the tool was developed by EU institutions and data are collected by government officials, while WHO and researchers take on a supporting role in validating and analysing the data. Consequently, the direct output of the HEPA Monitoring Framework—the PA Country Factsheets [65]—are a purely government-driven document; however, the scientific publications included in this review were classified as being based on a mainly government-driven, co-production or mainly research-driven approach, depending on the methodology of the respective study.

### HEPA PAT: “co-production”

Three studies applied WHO’s HEPA PAT. The tool was designed to assess national policy approaches to PA and consists of 29 closed and open-ended questions that are structured in 10 sections such as leadership and partnerships, policy documents, and evaluation [66]. Its development was based on a literature search, a cross reference check with WHO’s Global Strategy on Diet, Physical Activity and Health, and a pilot study in seven European countries [18, 67, 68].

All studies in this category have a strong focus on national policies. In addition, some aspects of subnational level policies are addressed by single questions of the HEPA PAT. Even though government officials supported all studies actively, the level of government involvement differed: While government officials or representatives of national institutes led the policy audit in some countries [67], the government was involved in selected stages of the process in other countries [69, 70]. Consequently, the approaches to apply the HEPA PAT differ between ‘true’ co-production and mainly research-driven applications.

### Other

17 of the remaining 57 studies were based on 15 other tools for PA policy monitoring. Ten of these tools monitor policies from a general perspective, i.e. are not limited to a specific sector or setting. These tools are located at all levels of the political system and analyse national level policies—sometimes in a global (e.g., GoPA) or European (Eurostat) comparison—as well as state and local level policies (e.g., CAPLA-SANTÉ, ANGELO framework). Four tools focus on the school setting and analyse policies at national level (Worldwide Survey of the situation of physical education in schools), state level (SHPSS), or school district and school level (e.g., S-PAPA).

The 57 studies in this category mainly focus on national (63.2%) and regional (50.9%) policies. Almost all studies follow a purely research-driven (52.6%) or mainly research-driven (40.4%) approach; only four studies (7.0%) used a co-production approach.

### Comparison of government involvement

A more detailed comparative analysis of the 18 identified tools for PA policy monitoring shows that there are differences with regards to the involvement of the government in different stages of the study process: Government representatives were mainly involved in data collection (13 tools) and sometimes also in data verification (6 tools); the other stages of the study process were usually exclusively conducted by researchers. For three tools, the government was involved in developing the study design, and only for one tool in data analysis and data interpretation (Table 3).

**Table 3:** Government involvement in study process

Tool	Study design	Data collection	Data verification	Data analysis	Data interpretation
Report Card on Physical Activity for Children and Youth	–	(X)	(X)	–	–
EU/WHO HEPA Monitoring Framework	X	X	X	–	–
Health-Enhancing Physical Activity Policy Audit Tool (HEPA PAT)	–	X	(X)	(X)	(X)
Active Community Environments (ACE)	–	X	(X)	–	–
Global Observatory for Physical Activity (GoPA!)	–	(X)	(X)	–	–
Analysis Grid for Environments Linked to Obesity (ANGELO) Framework	–	–	–	–	–
Australian Systems Approach to Physical Activity (ASAPA) tool	–	X	X	–	–
CAPLA-Santé	X	X	–	–	–
Community Health Assessment and Group Evaluation (CHANGE)	–	X	–	–	–
Eurostat	X	X	X	X	X
Four cornerstones of a successful national policy framework	–	–	–	–	–
HARDWIRED	–	(X)	–	–	–
WHO NCD progress monitor report	–	–	–	–	–
School Health Policies and Programs Study (SHPPS)	–	X	–	–	–
School Physical Activity Policy Assessment (S-PAPA)	–	–	–	–	–
Wellness School Assessment Tool	–	(X)	–	–	–
Worldwide Survey of the situation of physical education in schools	–	X	–	–	–
Health Eating and Physical Activity (HEPS) inventory tool	–	–	–	–	–

*X*  government involvement (including supranational equivalents), *(X)*  partial government involvement (e.g., in selected countries or studies), –  no government involvement or no information available

## Discussion

This review provided a comprehensive overview about the methods of PA policy monitoring. The review also showed that there are several approaches of PA policy monitoring that differ with regard to the tools they utilize, the policy level and setting they analyse, and the level of government involvement. Studies were grouped into four categories: Report Cards on Physical Activity for Children and Youth (*n* = 47), HEPA Monitoring Framework (*n* = 5), HEPA PAT (*n* = 3), and other (*n* = 57).

Policy monitoring approaches differed in being (purely or mainly) research-driven, or (purely or mainly) government-driven, or applying a co-production approach. Each of these approaches seems to have its own strengths and weaknesses, and is related to different models of research-policy relations [24]. In addition, the three most frequently applied tools are associated with different (implicit) theories of change with regards to generating impact on policy-making.

Research-driven approaches have the advantage that they collect data in a highly standardized and systematic way. In addition, the absence or low level of government involvement allows for an exclusively academic interpretation of data independent of political interests (which potentially allows for extremely critical verdicts of national policy). However, purely research-driven approaches that rely exclusively on a (systematic) literature search might have an extreme bias if only English-language publications are included. Furthermore, accounting for political context is difficult in international studies when no government officials are involved. Mainly research-driven approaches that involve government officials into selected stages of the research process can overcome these drawbacks to some extent. The Report Card on Physical Activity for Children and Youth is an example for a research-driven approach. Even though the tool does not focus exclusively on policy monitoring, it includes an indicator on government. The implicit assumption seems to be to generate a policy impact by assigning grades to governments and communicating them to the policy community (“knowledge shapes politics”). Evidence from studies analysing the impact of report cards indicates that the Canadian government used the tool as a source of information and as a ‘barometer’ for monitoring PA for children and youth [71]. Other countries reported that the tool facilitated discussions between researchers and the government or even led to the involvement of researchers into policymaking [72]. However, it is also reported that government officials were concerned about the legitimacy of the grade on the government indicator [71]. Therefore, it remains unclear whether a research-driven grading process is also appropriate for the Report Card’s government indicator (i.e., policy monitoring) or whether this approach encounters more resistance of decision-makers.

Government-driven approaches benefit from the direct access of government officials to data on policies and from their intimate knowledge of the policy environment. Consequently, highly contextualized information is available. However, data might not be as detailed as when a research-driven approach is applied, as government officials might not engage in in-depth desk research. It is also likely that studies either remain more descriptive since government officials might hesitate to be too critical of their governments’ policies, or that officials will attempt to describe their government’s policies as more favourable as they are to stave of potential criticism. The HEPA Monitoring Framework is an example for a government driven approach, as the framework was developed by the European Commission—based on a recommendation of the Council of the European Union—and data are provided by national governments (“politics shape knowledge”). The implicit assumption might be that the regular monitoring of PA policies is an incentive for governments to develop additional policies in order to fulfil more and more indicators of the HEPA Monitoring Framework. A comparative analysis of the data collected in 2015 and 2018 showed that 17 out of 27 countries improved the number of accomplished indicators while five maintained a constant number of indicators [25]. However, it remains unclear whether the progress in policy-making was influenced by the activities related to the HEPA Monitoring Framework. It remains also unclear, if governments take a “gaming the system” approach and start rather small-scale initiatives serving the sole purpose of fulfilling various of the often rather crude indicators.

Co-production approaches seem to combine the benefits of government-driven and research-driven approaches, i.e., to collect in-depth data via desk research while still relying on the knowledge of government officials. They are based on a clear theory of change, as the co-production approach can “produce research findings that are more likely (…) relevant to and used by the end users” [73] and might—as a consequence—have a higher policy impact compared to science-driven approaches. However, co-production approaches are resource intensive and require strong commitment from both government officials and researchers. The HEPA PAT is an example for a tool that was designed to initiate co-production approaches. Bull et al. explicitly stated that the HEPA PAT is not only an instrument to facilitate the collection of data but also “to stimulate critical debate, greater awareness, a broader dialogue among relevant actors and a higher sense of ownership within countries at the national and local level” [18]. As a result, the HEPA PAT might be “a catalyst for improved collaboration on future policy development and implementation” [18]. By applying a co-production approach, the HEPA PAT is based on a well-established knowledge translation strategy at the nexus between public health policy, practice and research [73, 74].

In this context, it seems to be of particular importance to reflect on the quality of policy monitoring: In most cases, data cannot be collected exclusively by researchers (limited insider knowledge) or government officials (limited capacity) in order to gather and analyse the best available information on policies for PA promotion. It also has to be noted that assessing policies based on an expert consensus is highly subjective, and there is a need to apply more systematic approaches for an objective policy assessment. Such approaches have been developed, e.g. a scoring rubric for the government indicator of the Report Card on Physical Activity for Children and Youth that is based on the HEPA PAT and allows to generate a total percentage score according to a defined process [75]. Furthermore, a Physical Activity Environment Policy Index (PA-EPI) has been developed recently that allows to assess the extent of implementation of government policies and actions in comparison to examples of international best practice; it is conceptualized as a two-component ‘policy’ and ‘infrastructure support’ framework and comprises forty-five ‘good practice statements’ across eight policy domains (such as, for instance, education, healthcare and sport-for-all) and seven infrastructure support domains (such as leadership, governance, and health-in-all-policies) [22].

Approaches for PA policy monitoring also differ with regards to the level of capacity building they may yield for governmental institutions. A monitoring approach directly involving governments may have the drawbacks of being resource-intensive and, when policies are assessed, lacking objectivity. However, it has the advantage of raising awareness for PA promotion within governments and fostering understanding for issues such as, for instance, definitions, recommendations and measurement issues.

When researchers and policy-makers are in the position to suggest or choose a specific approach for policy monitoring, they should consider the strengths and weaknesses of each approach with regards to data quality and capacity building. In addition, it needs to be considered at what stage policy-making is in a particular country (at an early stage, the need for applying a co-production approach might be the highest). Furthermore, the cultural appropriateness of a particular approach needs to be taken into consideration: While some governments might be very open to approaches that assess policy-making, governments in other countries might be rather reluctant to support policy monitoring approaches that rate or grade their own work.

There are some limitations to this study. First, the overarching results for the total of 112 studies are strongly influenced by the 47 studies that were based on the Report Cards on Physical Activity for Children and Youth. If these studies were not taken into account, the share of studies that were part of regular approaches to policy monitoring would have been significantly lower (16.9% instead of 51.8%) as well as the share of studies that assessed policies (20.0% instead of 53.8%). Second, the level of detail of information on methodological aspects of the included studies differed. For instance, Report Card studies usually provided a very general description of their methodology and did not include specific information on the grading process for the government indicator (an exemption is [75]). A third aspect is that we might not have identified all studies on PA policy monitoring, e.g. due to limitations of the search term or because they have been published recently (e.g., [76, 77]). This limitation also includes additional PA policy monitoring tools that were published in other languages—e.g. the Japanese Local Area Policy Audit Tool (L-PAT) [78]—as well as tools that have not been described in a scientific publication yet, e.g. because they are part of government-driven policy monitoring such as the Finnish TEAviisari tool [79]. Finally, the identification of tools for PA policy monitoring was partly influenced by the fact whether researchers have framed their methodology as a new tool or not.

## Conclusions

A large number of publications reports on results from different approaches to monitor policies in PA promotion. However, authors often do not reflect on the methods of data collection and the strengths and weaknesses of different approaches. Increasing awareness will help to put existing work in context and improve future monitoring endeavours. Depending on the purpose and resources, either of the approaches may be appropriate, but authors should discuss the associated risks and benefits.

Future research should focus on analysing the interplay of policy monitoring and the policy-making process to better understand research-policy relations in the field of PA promotion. In addition, an integrative global policy monitoring system for PA promotion that combines different approaches might be beneficial in order to increase data comparability and to strengthen synergies between different approaches for PA policy monitoring. From an overarching perspective, a comparison of policy monitoring initiatives in public health would be highly interesting to identify overarching patterns and to learn more about research-policy relations in this field.

### Supplementary Information

**Additional file 1.** Search terms.

**Additional file 2.** List of included studies.

## Data Availability

All data generated or analysed during this study are included in this published article and its additional files.

## Abbreviations

HEPA: Health-enhancing physical activity
HEPA PAT: Health-Enhancing Physical Activity Policy Audit Tool
NCD: Non-communicable diseases
PA: Physical activity
PA-EPI: Physical Activity Environment Policy Index
WHO: World Health Organization
