# Supplementary material for: Research- vs. government-driven physical activity policy monitoring: a systematic review across different levels of government
Source: Health Res Policy Syst. 2023 Nov 27;21:124. doi: 10.1186/s12961-023-01068-5 (PMC10680174; doi:10.1186/s12961-023-01068-5)
Supplement: Supplementary file 1 — Additional file 1. Search terms. [file 12961_2023_1068_MOESM1_ESM.docx]

**Additional file 1: Search terms**

| **Database** | **Search area** | **Search term** | **No. of identified studies** |
| --- | --- | --- | --- |
| PubMed | Title & Abstract | ((Physical activity[Title/Abstract]) AND ((Policy[Title/Abstract]) OR (Policies[Title/Abstract]))) AND (((Compar*[Title/Abstract]) OR (Assess*[Title/Abstract])) OR (Monitor*[Title/Abstract])) | 2,544 |
| SportDiscus (EBSCO)  PsycInfo (EBSCO) | All fields | Physical activity  AND  Policy OR Policies  AND  Compar* OR Assess* OR Monitor*  FILTER: Academic journals | 2,415 |
| Scopus (Elsevier) | Title, abstract, keywords | ( TITLE-ABS-KEY ( "physical activity" ) AND TITLE-ABS-KEY ( policy OR policies ) AND TITLE-ABS-KEY ( compar* OR assess* OR monitor* ) )    FILTER: Articles, reviews | 4,539 |
| Web of Knowledge | Topic (title, abstract, author keywords, and Keywords Plus.) | Physical activity (Topic)  and  Policy OR Policies (Topic)  and  Compar* OR Assess* OR Monitor* (Topic)  FILTER: Articles, review articles | 3,465 |
