# Supplementary material for: Research- vs. government-driven physical activity policy monitoring: a systematic review across different levels of government
Source: Health Res Policy Syst. 2023 Nov 27;21:124. doi: 10.1186/s12961-023-01068-5 (PMC10680174; doi:10.1186/s12961-023-01068-5)
Supplement: Supplementary file 2 — Additional file 2. List of included studies. [file 12961_2023_1068_MOESM2_ESM.docx]

**Additional file 2: List of included studies**

*Report Card on Physical Activity for Children and Youth*

Abdeta C, Teklemariam Z, Deksisa A, Abera E, Ocansey R, Okely AD. Assessment of Physical Activity Indicators for Children and Youth in Ethiopia: Evidence from the Global Matrix 3.0 Study (2017-2018). Sports Med Open. 2019;5(55).

Adeniyi AF, Odukoya OO, Oyeyemi AL, Adedoyin RA, Ojo OS, Metseagharun E, et al. Results From Nigeria's 2016 Report Card on Physical Activity for Children and Youth. J Phys Act Health. 2016;13(11, Suppl 2):S231-S6.

Aguilar-Farias N, Cortinez-O'Ryan A, Sadarangani KP, Von Oetinger A, Leppe J, Valladares M, et al. Results From Chile's 2016 Report Card on Physical Activity for Children and Youth. Journal of Physical Activity & Health. 2016;13:S117-S23.

Aguilar-Farias N, Miranda-Marquez S, Martino-Fuentealba P, Sadarangani KP, Chandia-Poblete D, Mella-Garcia C, et al. 2018 Chilean Physical Activity Report Card for Children and Adolescents: Full Report and International Comparisons. J Phys Act Health. 2020;17(8):807-15.

Amornsriwatanakul A, Nakornkhet K, Katewongsa P, Choosakul C, Kaewmanee T, Konharn K, et al. Results From Thailand's 2016 Report Card on Physical Activity for Children and Youth. J Phys Act Health. 2016;13(11, Suppl 2):S291-S8.

Andrade S, Ochoa-Avilés A, Freire W, Romero-Sandoval N, Orellana D, Contreras T, et al. Results from Ecuador’s 2018 Report Card on Physical Activity for Children and Youth. Journal of Physical Activity & Health. 2018;15(Suppl 2):S344-S6.

Aubert S, Barnes JD, Aguilar-Farias N, Cardon G, Chang CK, Delisle Nyström C, et al. Report Card grades on the physical activity of children and youth comparing 30 very high Human Development Index countries. J Phys Act Health. 2018;15:S298-S314.

Aubert S, Barnes JD, Forse ML, Turner E, González SA, Kalinowski J, et al. The International Impact of the Active Healthy Kids Global Alliance Physical Activity Report Cards for Children and Youth. J Phys Act Health. 2019;16(9):679-97.

Barnes JD, Cameron C, Carson V, Chaput JP, Faulkner GE, Janson K, et al. Results From Canada's 2016 ParticipACTION Report Card on Physical Activity for Children and Youth. J Phys Act Health. 2016;13(11, Suppl 2):S110-S6.

Barnes JD, Colley RC, Borghese M, Janson K, Fink A, Tremblay MS. Results from the active healthy kids Canada 2012 report card on physical activity for children and youth. Paediatr Child Health. 2013;18(6):301-4.

Barnes JD, Colley RC, Tremblay MS. Results from the active healthy kids Canada 2011 report card on physical activity for children and youth. Appl Physiol Nutr Metab. 2012;37(4):793-7.

Burghard M, de Jong NB, Vlieger S, Takken T. 2017 Dutch Report Card+: Results From the First Physical Activity Report Card Plus for Dutch Youth With a Chronic Disease or Disability. Front Pediatr. 2018;6(122).

Burghard M, Knitel K, van Oost I, Tremblay MS, Takken T. Is our Youth Cycling to Health? Results From the Netherlands' 2016 Report Card on Physical Activity for Children and Youth. J Phys Act Health. 2016;13(11, Suppl 2):S218-S24.

Dentro KN, Beals K, Crouter SE, Eisenmann JC, McKenzie TL, Pate RR, et al. Results from the United states' 2014 report card on physical activity for children and youth. J Phys Act Health. 2014;11 (Suppl 1):S105-S12.

Draper CE, Tomaz SA, Bassett SH, Harbron J, Kruger HS, Micklesfield LK, et al. Results from the healthy active kids south africa 2018 report card. SAJCH S Afr J Child health. 2019;13(3):130-6.

Galaviz KI, Arroyo MA, González-Casanova I, Villalobos MF, Jáuregui A, Ulloa EJ, et al. Results From Mexico's 2016 Report Card on Physical Activity for Children and Youth. J Phys Act Health. 2016;13(11, Suppl 2):S206-S12.

González SA, Sarmiento OL, Cohen DD, Camargo DM, Correa JE, Páez DC, et al. Results from Colombia's 2014 Report Card on Physical Activity for Children and Youth. J Phys Act Health. 2014;11 (Suppl 1):S33-S44.

Gray CE, Barnes JD, Cowie Bonne J, Cameron C, Chaput JP, Faulkner G, et al. Results from Canada's 2014 Report Card on Physical Activity for Children and Youth. J Phys Act Health. 2014;11 (Suppl 1):S26-S32.

Harrington DM, Belton S, Coppinger T, Cullen M, Donnelly A, Dowd K, et al. Results from Ireland's 2014 Report Card on Physical Activity in Children and Youth. J Phys Act Health. 2014;11 (Suppl 1):S63-S8.

Herrera-Cuenca M, Méndez-Perez B, Morales VC, Martín-Rojo J, Tristan B, Bandy AT, et al. Results From Venezuela's 2016 Report Card on Physical Activity for Children and Youth. J Phys Act Health. 2016;13(11, Suppl 2):S314-S29.

Katzmarzyk PT, Denstel KD, Beals K, Bolling C, Wright C, Crouter SE, et al. Results from the United States of America's 2016 report card on physical activity for children and youth. J Phys Act Health. 2016;13(11):S307-S13.

Kruusamäe H, Kull M, Mooses K, Riso EM, Jürimäe J. Results From Estonia's 2016 Report Card on Physical Activity for Children and Youth. J Phys Act Health. 2016;13(11, Suppl 2):S150-S6.

Liu Y, Tang Y, Cao ZB, Chen PJ, Zhang JL, Zhu Z, et al. Results From Shanghai's (China) 2016 Report Card on Physical Activity for Children and Youth. J Phys Act Health. 2016;13(11, Suppl 2):S124-S8.

Liukkonen J, Jaakkola T, Kokko S, Gråstén A, Yli-Piipari S, Koski P, et al. Results from Finland's 2014 report card on physical activity for children and youth. J Phys Act Health. 2014;11:S51-S7.

Maddison R, Dale LP, Marsh S, LeBlanc AG, Oliver M. Results from New Zealand's 2014 report card on physical activity for children and youth. J Phys Act Health. 2014;11:S83-S7.

Manyanga T, Makaza D, Mahachi C, Mlalazi TF, Masocha V, Makoni P, et al. Results from Zimbabwe's 2016 report card on physical activity for children and youth. J Phys Act Health. 2016;13(11):S337-S42.

Mota J, MJ ES, Raimundo AM, Sardinha LB. Results From Portugal's 2016 Report Card on Physical Activity for Children and Youth. J Phys Act Health. 2016;13(11, Suppl 2):S242-S5.

Nyström CD, Larsson C, Ehrenblad B, Eneroth H, Eriksson U, Friberg M, et al. Results from Sweden's 2016 report card on physical activity for children and youth. J Phys Act Health. 2016;13(11):S284-S90.

Oh JW, Lee EY, Lim J, Lee SH, Jin YS, Song BK, et al. Results from South Korea's 2018 Report Card on physical activity for children and youth. J Exerc Sci Fit. 2019;17(1):26-33.

Prista A, Daca T, Tchonga F, Machava E, Macucule C, Ribeiro E. Results From the Mozambique 2016 Report Card on Physical Activity for Children and Adolescents. J Phys Act Health. 2016;13(11, Suppl 2):S213-S7.

Reilly JJ, Dick S, McNeill G, Tremblay MS. Results from Scotland's 2013 Report Card on Physical Activity for Children and Youth. J Phys Act Health. 2014;11(Suppl 1):S93-S7.

Reilly JJ, Johnstone A, McNeill G, Hughes AR. Results From Scotland's 2016 Report Card on Physical Activity for Children and Youth. J Phys Act Health. 2016;13(11, Suppl 2):S251-S5.

Roman-Viñas B, Marin J, Sánchez-López M, Aznar S, Leis R, Aparicio-Ugarriza R, et al. Results From Spain's 2016 Report Card on Physical Activity for Children and Youth. J Phys Act Health. 2016;13(11, Suppl 2):S279-S83.

Schranz N, Olds T, Cliff D, Davern M, Engelen L, Giles-Corti B, et al. Results from Australia's 2014 Report Card on physical activity for children and youth. J Phys Act Health. 2014;11(S1):S21-S5.

Sember V, Starc G, Jurak G, Golobič M, Kovač M, Samardžija PP, et al. Results From the Republic of Slovenia's 2016 Report Card on Physical Activity for Children and Youth. J Phys Act Health. 2016;13(11, Suppl 2):S256-S64.

Sharif R, Chong KH, Zakaria NH, Ong ML, Reilly JJ, Wong JE, et al. Results from Malaysia's 2016 report card on physical activity for children and adolescents. J Phys Act Health. 2016;13(11):S201-S5.

Song Y, Yang HI, Lee EY, Yu MS, Kang MJ, Kang HJ, et al. Results From South Korea's 2016 Report Card on Physical Activity for Children and Youth. J Phys Act Health. 2016;13(11, Suppl 2):S274-S8.

Standage M, Wilkie HJ, Jago R, Foster C, Goad MA, Cumming SP. Results From England's 2014 Report Card on Physical Activity for Children and Youth. Journal of Physical Activity & Health. 2014;11:S45-S50.

Sukys S, Emeljanovas A, Gruodyte-Raciene R, Mieziene B, Trinkuniene L, Rutkauskaite R, et al. Results from Lithuania's 2018 Report Card on Physical Activity for Children and Youth. Int J Environ Res Public Health. 2019;16(4710).

Takken T, de Jong N, Duijf M, van den Berg S, Wendel-Vos W, Dutch Physical Activity Report Card Study G. Results from the Netherlands' 2018 Report Card and Report Card+ on physical activity for children and youth with and without chronic medical condition. Public Health. 2020;185:161-6.

Tammelin TH, Aira A, Hakamäki M, Husu P, Kallio J, Kokko S, et al. Results From Finland's 2016 Report Card on Physical Activity for Children and Youth. J Phys Act Health. 2016;13(11, Suppl 2):S157-S64.

Tanaka C, Tanaka S, Inoue S, Miyachi M, Suzuki K, Abe T, et al. Results from the Japan's 2018 report card on physical activity for children and youth. J Exerc Sci Fit. 2019;17(1):20-5.

Tyler R, Mannello M, Mattingley R, Roberts C, Sage R, Taylor SR, et al. Results from Wales' 2016 report card on physical activity for children and youth: Is Wales turning the tide on children's inactivity? J Phys Act Health. 2016;13(11):S330-S6.

Uys M, Bassett S, Draper CE, Micklesfield L, Monyeki A, De Villiers A, et al. Results from South Africa's 2016 report card on physical activity for children and youth. J Phys Act Health. 2016;13(11):S265-S73.

Wachira LJM, Muthuri SK, Tremblay MS, Onywera VO. Results from Kenya's 2014 report card on the physical activity and body weight of children and youth. J Phys Act Health. 2014;11(S1):S69-S73.

Ward MR, Tyler R, Edwards LC, Miller MC, Williams S, Stratton G. The AHK-Wales Report Card 2018: Policy Measures - is it possible to 'score' qualitative data? Health Promot Int. 2020;36(4):1151-9.

Wilkie H, Standage M, Sherar L, Cumming S, Parnell C, Davis A, et al. Results From England's 2016 Report Card on Physical Activity for Children and Youth. J Phys Act Health. 2016;13(11, Suppl 2):S143-S9.

*HEPA Monitoring Framework*

Breda J, Jakovljevic J, Rathmes G, Mendes R, Fontaine O, Hollmann S, et al. Promoting health-enhancing physical activity in Europe: Current state of surveillance, policy development and implementation. Health Policy. 2018;122(5):519-27.

Gelius P, Tcymbal A, Abu-Omar K, Mendes R, Tribuzi Morais S, Whiting S, et al. Status and contents of physical activity recommendations in European Union countries: a systematic comparative analysis. BMJ Open. 2020;10(2).

Mutz M, van Munster M. Associations of Physical Activity Policies With Sports Participation in EU Countries: Higher Overall Levels, Smaller Social Inequalities, and More Positive Trends Since 2009. J Phys Act Health. 2020;17(4):464-70.

Tcymbal A, Gelius P, Abu-Omar K, Foster C, Whiting S, Mendes R, et al. Development of national physical activity recommendations in 18 EU member states: a comparison of methodologies and the use of evidence. BMJ Open. 2021;11(4).

Whiting S, Mendes R, Morais ST, Gelius P, Abu-Omar K, Nash L, et al. Promoting health-enhancing physical activity in Europe: Surveillance, policy development and implementation 2015-2018. Health Policy. 2021;125(8):1023-30.

*HEPA PAT*

Bull FC, Milton K, Kahlmeier S, Arlotti A, Juričan AB, Belander O, et al. Turning the tide: national policy approaches to increasing physical activity in seven European countries. Br J Sports Med. 2015;49(11):749-56.

Gelius P, Messing S, Forberger S, Lakerveld J, Mansergh F, Wendel-Vos W, et al. The added value of using the HEPA PAT for physical activity policy monitoring: a four-country comparison. Health Res Policy Syst. 2021;19(22).

Van Hoye A, Vandoorne C, Absil G, Lecomte F, Fallon C, Lombrail P, et al. Health enhancing physical activity in all policies? Comparison of national public actors between France and Belgium. Health Policy. 2019;123(3):327-32.

*Other*

Allen LN, Nicholson BD, Yeung BYT, Goiana-da-Silva F. Implementation of non-communicable disease policies: a geopolitical analysis of 151 countries. Lancet Glob Health. 2020;8(1):e50-e8.

Anderson L, Foster S, Flynn R, Fitterman M. Assessing public policies and assets that affect obesity risk while building new public health partnerships, New Hampshire, 2011. Prev Chronic Dis. 2013;10.

Bellew B, Schöeppe S, Bull FC, Bauman A. The rise and fall of Australian physical activity policy 1996 - 2006: a national review framed in an international context. Aust New Zealand Health Policy. 2008;5(18).

Benjamin-Neelon SE, Neelon B, Pearce J, Grossman ER, Gonzalez-Nahm S, Slining M, et al. State Regulations Promoting Infant Physical Activity in Early Care and Education. Child Obes. 2018;14(6):368-74.

Boehm R, Schwartz MB, Lowenfels A, Brissette I, Pattison MJ, Ren J. The Relationship between Written District Policies and School Practices among High-Need Districts in New York State. J Sch Health. 2020;90(6):465-73.

Bornstein DB, Pate RR, Pratt M. A review of the national physical activity plans of six countries. J Phys Act Health. 2009;6(s2, Suppl 2):S245-S64.

Brennan LK, Kemner AL, Donaldson K, Brownson RC. Evaluating the implementation and impact of policy, practice, and environmental changes to prevent childhood obesity in 49 diverse communities. J Public Health Manag Pract. 2015;21 (Suppl 3):S121-S34.

Bullen C, Lyne M. A survey of New Zealand's Territorial Local Authorities' policies, plans and programs promoting physical activity. Aust N Z J Public Health. 2006;30(4):334-6.

Carlson JA, Sallis JF, Chriqui JF, Schneider L, McDermid LC, Agron P. State policies about physical activity minutes in physical education or during school. J Sch Health. 2013;83(3):150-6.

Ceccarelli A. Review of policies adopted in 34 countries to improve diet and physical activity. Ital J Public Health. 2011;8(2):156-71.

Chalkley A, Milton K. A critical review of national physical activity policies relating to children and young people in England. J Sport Health Sci. 2021;10(3):255-62.

Chriqui JF, Leider J, Temkin D, Piekarz-Porter E, Schermbeck RM, Stuart-Cassel V. State Laws Matter When It Comes to District Policymaking Relative to the Whole School, Whole Community, Whole Child Framework. J Sch Health. 2020;90(12):907-17.

Christiansen NV, Kahlmeier S, Racioppi F. Sport promotion policies in the European Union: results of a contents analysis. Scand J Med Sci Sports. 2014;24(2):428-38.

Cradock ALI, O'Donnell EM, Benjamin SE, Walker E, Slining M. A review of state regulations to promote physical activity and safety on playgrounds in child care centers and family child care homes. J Phys Act Health. 2010;7(Suppl 1):S108-S19.

Crombie IK, Irvine L, Elliott L, Wallace H. Targets to tackle the obesity epidemic: a review of twelve developed countries. Public Health Nutr. 2009;12(3):406-13.

Darfour-Oduro SA, Andrade JE, Grigsby-Toussaint DS. Review of policies to increase fruit and vegetable consumption and physical activity in 49 low- and middle-income countries. J Public Health (Oxf). 2019;41(1):119-29.

Dolmatova TV, Zubkova AV. ОСНОВНЫЕ ТЕНДЕНЦИИ РЕГУЛИРОВАНИЯ В СФЕРЕ ФИЗИЧЕСКОЙ АКТИВНОСТИ И СПОРТА ЗА РУБЕЖОМ: АНАЛИЗ ВЕДУЩИХ ЗАРУБЕЖНЫХ ПРАКТИК [Key trends in regulating physical activity and public sport: Analysis of successful foreigh practice]. Human Sport Med. 2021;21(1):108-16.

Duffey KJ, Slining MM, Benjamin Neelon SE. States lack physical activity policies in child care that are consistent with national recommendations. Child Obes. 2014;10(6):491-500.

Esdaile E, Thow AM, Gill T, Sacks G, Golley R, Love P, et al. National policies to prevent obesity in early childhood: Using policy mapping to compare policy lessons for Australia with six developed countries. Obes Rev. 2019;20(11):1542-56.

Gołdys AM. Sport sector as a part of public policy for elderly people in selected EU countries. Epidemiol Biostat Public Health. 2017;14(2).

Graham-DeMello A, Yusuf J, Kay-Arora M, Hancock Friesen CL, Kirk SFL. Understanding the Environment for Health-Promoting Schools Policies in Nova Scotia: A Comprehensive Scan at the Provincial and Regional School Level. Int J Environ Res Public Health. 2021;18(7).

Hämäläinen RM, Sandu P, Syed AM, Jakobsen MW. An evaluation of equity and equality in physical activity policies in four European countries. Int J Equity Health. 2016;15(191).

Hardman K. Physical education in schools: A global perspective. Kinesiology. 2008;40(1):5-28.

Hassan OB, Herman KM, Kryzanowski CD, Faulkner GEJ. Active living in Saskatchewan: A review of official community plans. Can J Public Health. 2018;108(5-6):e551-e7.

Heinrich KM, Johnson CB, Jokura Y, Nett B, Maddock JE. A survey of policies and local ordinances supporting physical activity in Hawaii counties. Prev Chronic Dis. 2008;5(1).

Kahlmeier S, Wijnhoven TMA, Alpiger P, Schweizer C, Breda J, Martin BW. National physical activity recommendations: systematic overview and analysis of the situation in European countries. BMC Public Health. 2015;15(133).

Klepac Pogrmilovic B, Ramirez Varela A, Pratt M, Milton K, Bauman A, Biddle SJH, et al. National physical activity and sedentary behaviour policies in 76 countries: availability, comprehensiveness, implementation, and effectiveness. Int J Behav Nutr Phys Act. 2020;17(116).

Król U. Taxes as instruments of health-related sports policy in European countries. Journal of Physical Education & Sport. 2020;20:1038-43.

Lachat C, Otchere S, Roberfroid D, Abdulai A, Seret FM, Milesevic J, et al. Diet and physical activity for the prevention of noncommunicable diseases in low- and middle-income countries: a systematic policy review. PLoS Med. 2013;10(6).

Librett JJ, Yore MM, Schmid TL. Local ordinances that promote physical activity: a survey of municipal policies. Am J Public Health. 2003;93(9):1399-403.

Lillehoj CJ, Daniel-Ulloa JD, Nothwehr F. Prevalence of Physical Activity Policies and Environmental Strategies in Communities and Worksites: The Iowa Community Transformation Grant. J Occup Environ Med. 2016;58(1):e1-e5.

Lounsbery MAF, McKenzie TL, Morrow JR, Monnat SM, Holt KA. District and school physical education policies: Implications for physical education and recess time. Ann Behav Med. 2013;45(Suppl 1):S131-S41.

Lowe M, Arundel J, Hooper P, Rozek J, Higgs C, Roberts R, et al. Liveability aspirations and realities: Implementation of urban policies designed to create healthy cities in Australia. SOCIAL SCIENCE & MEDICINE. 2020;245.

Lozzi G, Monachino MS. Health considerations in active travel policies: A policy analysis at the EU level and of four member countries. Research in Transportation Economics. 2021;86.

Maximova K, Raine KD, Czoli C, O'Loughlin J, Minkley J, Tisdale K, et al. Monitoring progress toward United Nations commitments: characteristics of Canadian legislation to promote tobacco control, physical activity and healthy eating. A descriptive study. CMAJ Open. 2019;7(4):E745-E53.

Milton K, Bauman A. A critical analysis of the cycles of physical activity policy in England. Int J Behav Nutr Phys Act. 2015;12(8).

Mooney JD, Jepson R, Frank J, Geddes R. Obesity Prevention in Scotland: A Policy Analysis Using the ANGELO Framework. Obes Facts. 2015;8(4):273-81.

Moore LV, Carlson SA, Onufrak S, Carroll DD, Galuska D. Development and implementation of a local government survey to measure community supports for healthy eating and active living. Preventive Med Reports. 2017;6:74-9.

Murphy MM, Unwin N, Samuels TA, Hassel TA, Bishop L, Guell C. Evaluating policy responses to noncommunicable diseases in seven Caribbean countries: challenges to addressing unhealthy diets and physical inactivity. Rev Panam Salud Publica. 2018;42.

Musingarimi P. Obesity in the UK: A review and comparative analysis of policies within the devolved administrations. Health Policy. 2009;91(1):10-6.

Nanney MS, Nelson T, Wall M, Haddad T, Kubik M, Laska MN, et al. State school nutrition and physical activity policy environments and youth obesity. Am J Prev Med. 2010;38(1):9-16.

Nau T, Lee K, Smith BJ, Bellew W, Reece L, Gelius P, et al. Toward Whole-of-System Action to Promote Physical Activity: A Cross-Sectoral Analysis of Physical Activity Policy in Australia. J Phys Act Health. 2019;16(11):1029-38.

Nethe A, Dorgelo A, Kugelberg S, van Assche J, Buijs G, Yngve A, et al. Existing policies, regulation, legislation and ongoing health promotion activities related to physical activity and nutrition in pre-primary education settings: An overview. Obes Rev. 2012;13(Suppl 1):118-28.

Nova J. Using the concept of sports business intelligence in evaluating sport policies. Sport Mont. 2018;16(2):73-6.

Parrish A-M, Tremblay MS, Carson S, Veldman SLC, Cliff D, Vella S, et al. Comparing and assessing physical activity guidelines for children and adolescents: a systematic literature review and analysis. International Journal of Behavioral Nutrition and Physical Activity. 2020;17(16).

Pate RR, Frongillo EA, McIver KL, Colabianchi N, Wilson DK, Collie-Akers VL, et al. Associations between community programmes and policies and children's physical activity: the Healthy Communities Study. Pediatr Obes. 2018;13:72-81.

Pate RR, Trilk JL, Byun W, Wang J. Policies to Increase Physical Activity in Children and Youth. Journal of Exercise Science & Fitness. 2011;9(1):1-14.

Racine AN, Garbarino JM, Massiera B, Vuillemin A. Analysis of the local health-enhancing physical activity policies on the french riviera. Int J Environ Res Public Health. 2021;18(156).

Rütten A, Frahsa A, Engbers L, Gusi N, Mota J, Pacenka R, et al. Supportive environments for physical activity, community action, and policy in 8 European Union Member States: comparative analysis and specificities of context. J Phys Act Health. 2014;11(5):873-83.

Seppälä T, Hankonen N, Korkiakangas E, Ruusuvuori J, Laitinen J. National policies for the promotion of physical activity and healthy nutrition in the workplace context: a behaviour change wheel guided content analysis of policy papers in Finland. BMC Public Health. 2017;18(87).

Slining MM, Benjamin Neelon SE, Duffey KJ. A review of state regulations to promote infant physical activity in child care. International Journal of Behavioral Nutrition and Physical Activity. 2014;11(139).

Smith EM, Wilburn G, Estabrooks PA. The quality of school physical activity policies within Maryland and Virginia. Journal of Physical Activity & Health. 2015;12(4):500-5.

Spitters HP, Lau CJ, Sandu P, Quanjel M, Dulf D, Glümer C, et al. Unravelling networks in local public health policymaking in three European countries - a systems analysis. Health Res Policy Syst. 2017;15(5).

Sreedhara M, Goulding M, Valentine Goins K, Frisard C, Lemon SC. Healthy Eating and Physical Activity Policy, Systems, and Environmental Strategies: A Content Analysis of Community Health Improvement Plans. Front Public Health. 2020;8(580175).

Szczepaniak M. Public sport policies and health: Comparative analysis across European union countries. J Phys Educ Sport. 2020;20:1022-30.

Tuangratananon T, Wangmo S, Widanapathirana N, Pongutta S, Viriyathorn S, Patcharanarumol W, et al. Implementation of national action plans on noncommunicable diseases, Bhutan, Cambodia, Indonesia, Philippines, Sri Lanka, Thailand and Viet Nam. Bull World Health Organ. 2019;97(2):129-41.

Varela AR, Pratt M, Powell K, Lee IM, Bauman A, Heath G, et al. Worldwide surveillance, policy, and research on physical activity and health: The global observatory for physical activity. J Phys Act Health. 2017;14(9):701-9.
